# Development of a Codebook of Online Anti-Vaccination Rhetoric to Manage COVID-19 Vaccine Misinformation

**DOI:** 10.3390/ijerph18147556

**Published:** 2021-07-15

**Authors:** Brian Hughes, Cynthia Miller-Idriss, Rachael Piltch-Loeb, Beth Goldberg, Kesa White, Meili Criezis, Elena Savoia

**Affiliations:** 1 Polarization and Extremism Research and Innovation Lab (PERIL), American University, Washington, DC 20016, USA; cynthia@american.edu (C.M.-I.); kw4947a@american.edu (K.W.); criezis@american.edu (M.C.); 2 Program of Justice, Law, and Criminology, School of Public Affairs, American University, Washington, DC 20016, USA; 3 Emergency Preparedness Research Evaluation & Practice (EPREP) Program, Division of Policy Translation & Leadership Development, Harvard T.H. Chan School of Public Health, Boston, MA 02115, USA; piltch-loeb@hsph.harvard.edu; 4 Jigsaw, Google LLC, New York, NY 10011, USA; bethgoldberg@google.com; 5 Emergency Preparedness Research Evaluation & Practice (EPREP) Program, Harvard T.H. Chan School of Public Health, Boston, MA 02115, USA; esavoia@hsph.harvard.edu

**Keywords:** COVID-19, vaccine hesitancy, anti-vax, public health communication, attitudinal inoculation, misinformation, disinformation

## Abstract

Vaccine hesitancy (delay in obtaining a vaccine, despite availability) represents a significant hurdle to managing the COVID-19 pandemic. Vaccine hesitancy is in part related to the prevalence of anti-vaccine misinformation and disinformation, which are spread through social media and user-generated content platforms. This study uses qualitative coding methodology to identify salient narratives and rhetorical styles common to anti-vaccine and COVID-denialist media. It organizes these narratives and rhetorics according to theme, imagined antagonist, and frequency. Most frequent were narratives centered on “corrupt elites” and rhetorics appealing to the vulnerability of children. The identification of these narratives and rhetorics may assist in developing effective public health messaging campaigns, since narrative and emotion have demonstrated persuasive effectiveness in other public health communication settings.

## 1. Introduction

The COVID-19 pandemic has arisen in a period of increasing vaccine hesitancy in the United States. Vaccine hesitancy is defined as a delay in the acceptance of a vaccine or the outright refusal to take a vaccine despite its availability [1] Vaccine hesitancy is of particular concern when a vaccine is the primary method to mitigate the spread of a serious disease. Prior to the beginning of the COVID-19 pandemic, vaccine hesitancy was declared one of the top ten threats to global health by the World Health Organization [2]. The pandemic emerged shortly after a 2019 measles outbreak, which has since been tied to parental reluctance to vaccinate schoolchildren [3,4]. This was the worst outbreak of its kind since 1992 [5] and since a historic low of 0.15 measles cases per million in a 2002 [6] outbreak.

Several national opinion polls have found a significant portion of the US population is hesitant to take a COVID-19 vaccine. The prevalence of COVID-19 vaccine hesitancy has ranged from a quarter to half of the US population depending on the time in which surveys were conducted, reflecting the ongoing challenge of addressing vaccine hesitancy in the pursuit of herd immunity [6,7].

### 1.1. The Role of Online Fora and Social Media

Multiple mechanisms can contribute to the spread of vaccine hesitancy, among which online forums play an important role in persuasion [8,9,10]. In the past, persuasion might have occurred via a “two-step flow,” in which opinion leaders were integral to the adoption of people’s worldviews [11]. However, with the growth of increasingly personalized and automatically customized content channels offered on the web, some scholars have observed the emergence of a complementary “one-step flow” of persuasion [12,13]. In this “one-step flow,” web users are influenced directly from an online experience that has been algorithmically tailored to address their interests—and psychological vulnerabilities [14]. The present study is informed by the hybrid theory of Hilbert et al. [15], which argues that while persuasion has indeed become less interpersonal, and more automated and direct, online influencers and trusted offline sources still play a crucial role in shaping opinion.

Several authors have examined how social media platforms contributed to vaccine hesitancy prior to the COVID-19 outbreak [16,17,18,19,20]. Amplification of vaccine hesitancy online has continued in light of development of the COVID-19 vaccine [21]. Studies reviewed by Puri and colleagues [21] describe how anti-vaccine content frequently generates greater user engagement than its pro-vaccine counterparts on Facebook. Singh and colleagues [22] found that low-quality sources of misinformation on COVID-19 were more commonly retweeted than those with high-quality information. In a comparative analysis of the spread of misinformation on five social media platforms (Twitter, Instagram, YouTube, Reddit, and Gab), Cinelli and colleagues [23] analyzed more than 8 million comments and posts over a time span of 45 days, to model the spread of misinformation and demonstrated that social media platforms can serve as amplifiers of misinformation.

Vaccine hesitancy at large often arises in response to real-world events that create a window of opportunity for narratives countering vaccine uptake. According to Betsch et al, even limited and short-term exposure to anti-vaccine websites increased individual perceptions of vaccine risks [24]. Buller and colleagues [25] have found that an individual’s degree of engagement with Facebook posts on the HPV vaccine was predictive of greater vaccine hesitancy. Buller et al. hypothesize that on the social media platform, alleged risks associated with vaccines appear more immediate and tangible than risks associated with not receiving the vaccine (the success of vaccination is the absence of disease) [21]. Further, anti-vaccine messaging tends to be more focused on emotions and personal anecdotes with powerful imagery in contrast to the empirical strategies utilized by pro-vaccination literature and platforms. These emotional approaches tend to be more appealing to social media users and are consistent with other content that tends to be shared on social media [26].

### 1.2. Types of Anti-Vax Narratives and Beliefs

Often vaccine hesitancy is treated by policymakers and risk communicators as a uniform belief; people are either hesitant to receive a vaccine or not hesitant. However, a subset of research has identified ways in which vaccine hesitancy is not a uniform belief system, but rather consists of several tropes and narratives that coalesce around common themes. In a review of 480 websites, all of which were promoting vaccine hesitancy, Moran and colleagues [20] found several key values among those reviewed: choice, freedom, natural/holism, independence/individuality, and religion. These values were expressed in narratives related to topics ranging from lifestyle norms (references to alternative messaging or other health behaviors), mistrust in communicating authority, vaccine-related diseases, and vaccine ingredients counter to religion (pork products, for example). The most common value was “choice,” appearing on one-third of the sample identified in the study.

Moran was the first to consider values or the “core” principles that may drive the more specific beliefs held by—or comments made by—individuals. The study by Moran and colleagues adds to an ongoing literature aiming to characterize the type of anti-vaccination content on the internet [6,27,28,29]. These prior studies have identified a variety of specific comments or beliefs among content producers. Bean [27] builds on the work of Davies and Wolfe and refers to specific values that emerge from this content: safety and effectiveness, civil liberties, and alternative treatments. More recent work by Johnson et al. [30] has found that there is a greater number of smaller anti-vaccination groups online relative to pro-vaccination groups, allowing these groups to cover more “surface area” and attract the undecided to anti-vaccination messages. This “surface area” is further expanded when anti-vaccination groups create content focused on more than just vaccination-related issues, connecting to the other core beliefs these authors have described. Critically, Johnson et al. note that anti-vaccine messages tend to be framed as conversations between equals, that is, as peer-to-peer communication. Conversely, pro-vaccine content tends to take the form of expert-to-masses, top-down messages. Anti-vaccine communication, thus, bridges the gap between one-step and two-step flows of persuasion, perhaps the better to take advantage of online media’s unique style of social ambiguity.

### 1.3. Defining Rhetoric and Narrative in the Context of Ant-Vaccine and COVID-Denial Content

Whereas these prior studies analyzed the quality of information related to vaccine hesitancy and the values that motivate hesitancy, the study we conducted focuses on the narrative tropes and rhetorical styles of anti-vaccine content. Narratives combined with particular rhetorical strategies can be more or less influential. This study adopts a simple definition of the term “narrative” predicated on the features of change and temporality. Dahlstrom provides an excellent definition of narrative in the context of science communication to mass audiences as “a particular structure that describes the cause-and-effect relationships that take place over a particular time period that impact particular characters” [31] (p. 13614)**.** Our simple definition is in keeping with established scholarly uses of the term [32,33,34,35].

For the purposes of this study, a “trope” is understood to mean a subsidiary element of narrative, indicative of the larger narrative or narratives in which it is contained [32,36,37,38]—narratives which might be vague, only partially articulated, or even based on mysterious/absent plot, characters, and/or meaning [39,40,41]. Tropes, therefore, “are about relationships, and never about the term in itself” [42] (p. 3). Tropes, in other words, definitionally refer to something beyond the context in which they appear. A “narrative trope,” therefore, is some semiotic element—visual, aural, or written—which connotes a larger story or worldview.

For the purposes of this study, a “rhetorical strategy” refers to those modes of persuasion that can be separated from narrative appeals, which do not in-and-of-themselves indicate a larger story or narrativized worldview. These generally comport with the classical categories of rhetorical appeal—ethos (authority), pathos (emotion), and logos (logic) [43]—and with the Platonic critique of rhetoric such as vulgar and manipulative [44]. On one hand, we look to the classical definitions because contemporary definitions of rhetoric are diverse and unresolved [45,46,47,48]. This study takes an expansive view of rhetoric, to include audio-visual as well as written and verbal rhetoric. This is in keeping with the common scholarly use of the terms and concept [49,50,51,52]. Narrative tropes and rhetorical strategies, thus, combine to form persuasive messages, which may be either anti-social (as in this study) or prosocial (as in the public health messaging described below).

### 1.4. The Efficacy of Narrative and Rhetorical Persuasion vs. Appeals to Reason

While the literature is not uniform in its conclusions [53,54], there is evidence to suggest that narrative and perspectival persuasion can be more effective than factual–argumentative approaches [55,56,57,58,59,60,61,62,63]. Research shows that audience absorption into a narrative reduces the audiences’ capacity to form counterarguments against messages contained in the narrative [64,65,66] and tends to strengthen the persuasiveness of weak arguments and appeals [67]. As per Maertens, Roozenbeek and Van der Linden [68], messages addressing a persuasion technique rather than a specific factual claim offer the potential “to achieve broad-spectrum resistance against manipulation,” which factual rebuttal might not [68] (n.p.).

Braddock’s work in the field of attitudinal inoculation likewise suggests that attention to form yields greater negative attitudinal reactance against undesirable belief or behavior than attention to content [69]. Ratcliff and Sun argue that narrative’s effectiveness comes from its ability to circumvent psychological resistance, so that “when a persuasive message is embedded in the story and/or carried by the characters, persuasion occurs to the immersed, less critical, and less defensive” [52] (p. 414). Additionally, literature from the field of violent extremism studies suggests that individuals are most often brought to false and conspiratorial perspectives not by rational decision making, but through a search for emotional and social fulfillment [70,71].

Work in the field of public health messaging has demonstrated that messaging that focuses on narrative and rhetoric (form) tends to yield better persuasive outcomes than messaging that focuses on facts alone (i.e., content) [55,60,62,72,73,74]. A variety of studies from the field of advertising, media, and communication studies suggest that narrative messaging yields more effective outcomes than analytic and informative messages [66,67,75]. As a practical matter, Deng et al. found advertising messaging related to COVID-19 deployed “transformational” strategies (that is, appealing to emotions) over informational ones by a ratio of nearly two-to-one [76].

### 1.5. Study Objective

In anticipation of the unfolding COVID-19 vaccination campaign, this study sought to identify narrative tropes and rhetorical strategies in the emerging vaccine hesitancy discourse. To this end, we developed a codebook of the most commonly found anti-vaccination themes, both general and specific to COVID-19. We did so with the intent of providing public health communicators and social media moderation teams with a codebook of compelling anti-vaccination themes, against which counter-messages might be tailored. This work contributes to the nascent literature on COVID-19 vaccine hesitancy beliefs and the ways in which anti-vaccine messages are persuasive.

## 2. Materials and Methods

### 2.1. Manual Coding Methodology

This study employs a simple manual coding approach to identify the narrative tropes and rhetorical styles described above. This method uses both inductive and deductive analytic approaches to gather its corpora. These corpora are analyzed both inductively and deductively, in two rounds of independent manual coding by a team of three subject area experts. Each team member’s codes are compared so as to substantiate their relevance, and then consolidated “to construct more abstract concepts” [77] (p. 2) in keeping with the action research approach of identifying salient narratives and rhetorics. This final body of codes is then assessed quantitatively to determine code frequency and distribution, that is, both how many times a code appeared and how many distinct pieces of content it appeared in. This methodological approach is a conventional and widely accepted tool [78,79,80,81], which excels at identifying “summative, salient, essence-capturing, and/or evocative attribute[s]” [80] (p. 4) in mixed-media corpora. This subtlety and complexity is not so easily gleaned using more quantitatively centered methods and large datasets. However, similar to any method, it does possess shortcomings. These gaps, and the future avenues of study they suggest, are detailed in the “Future Directions” section at the end of this article.

### 2.2. Data Sources

Data collection aimed to develop corpora (i.e., large collections of codable content) for two separate rounds of coding. These corpora were developed via purposeful sampling methodology. Using hashtag and keyword searchers, a team of subject matter experts identified 20 channels (i.e., bounded sources of content, such as a social media account), which appeared to contain a high degree of anti-vaccine content and/or COVID denialism. For each round, five distinct channels were selected for preliminary coding. Only publicly available, English language media were selected, both to comply with platform terms of service and to focus coding efforts on content available to the uncommitted and vaccine hesitant. Geography was not considered, due to the transnational availability of the channels that were surveyed. Channels were selected according to the criteria of (1) offering 50–100 pieces of codable content from the past six months, (2) covering written, video, and/or image/meme media, (3) related to COVID skepticism/denial, anti-vaccine ideology, and/or conspiracism, and (4) discursive significance, based on quantitative and qualitative evaluation.

The five corpora coded in this first round included: the *Plandemic* propaganda documentary, the *Vaxxed* propaganda documentary, official posts from the Facebook group of the anti-vaccine and COVID-skeptic organization The Children’s Health Defense (going back three months, excluding comments), the “fake news” blog *OffGuardian*’s “COVID Factchecker” vertical (comprising seven blog posts), and the Instagram account “COVID Funny Memes” (constituting 100 pieces of relevant content, excluding comments). The discursive significance of these channels was established based on the following criteria: the films *Vaxxed* and *Plandemic* enjoyed widespread distribution on online platforms [82,83]; the Children’s Health Defense organization’s Facebook page has over 145,000 followers and is headed by celebrity vaccine detractor Robert F. Kennedy Jr. [84]; the “Funny COVID Memes” page had only approximately 5000 followers, but was selected for its apparent ideological neutrality (i.e., both vaccine-denialist and pro-vaccine content appeared in roughly equal proportion); the *OffGuardian* blog represented the least widely consumed inclusion, ranking 74,278 in global internet engagement according to *Alexa* rankings—it was chosen in order to include a text-only coding source and due to its clear COVID/vaccine-skeptic position.

Sampling for the second round was likewise purposive, with a focus on capturing media and audiences that might have been missed during Round 1. Channels that spoke to minority demographics were specifically sought out. Once again, five channels were selected for coding based on the same criteria as Round 1. The five corpora constituting the second round of coding were: the Twitter account of Joyce Brooks (a representative of the Denver NAACP and vaccine denialist), the Twitter account of Toby Rogers (a popular and prolific anti-vaccine figure with a following of 29.7k at the time of sampling), the YouTube video “A Message to Aussie Muslims” by Sufyaan Khalifa (urging resistance to COVID public health measures), the Gardasil Girls “vaccine injury” Instagram account (including top comments), and the Vaccines Uncovered Instagram account. For all four non-video accounts, 50 individual posts, including retweets and reposts, were collected.

The purpose of this selection methodology was not to select the most popular or influential COVID/vaccine-skeptic/denialist channels, but to select a spread of channels that could reasonably be expected to move the research team toward theoretical saturation. Saturation was defined by the research team as that point at which “additional data do not lead to any new emergent themes” [85] (p. 135).

### 2.3. Coding Methodology

Round 1 corpora were loaded into NVivo, and each member of the four-person codebook team independently coded each corpora. Codes were then compared and consolidated. Infrequent codes were set aside. In the end, preliminary coding produced 34 unique codes pertaining to the narrative tropes and rhetorical strategies that circulate in the anti-vaccine and COVID-skeptic media.

These initial 34 codes were then applied to the second round of corpora. Round 2 corpora were coded according to the preliminary codebook produced in Round 1, with new codes added as deemed appropriate by the coding team. The coding schema developed by each individual member of the codebook team were compared and consolidated. An additional 29 codes were identified during the second round of coding. However, these codes were either found only sporadically or could be consolidated into existing codes. These codes were then analyzed according to an action research methodology approach [86]. The rates of code appearance across corpora were analyzed in comparison with their frequency within corpora. Based on repetition of themes, it was determined that codes pertaining to general audiences (i.e., not demographically specific) had reached a point of adequate saturation. However, in keeping with action research methodology, some relatively infrequent codes were kept. This was done in order to address codes specific to minority demographics.

The codebook team drafted detailed explanations of the codes and then grouped them according to whether they referred to a narrative trope or a rhetorical strategy, based on the criteria outlined in the literature review. Narrative tropes were further organized according to the implicit antagonist of the narrative. Implicit antagonists were determined based on close reading of content from which these codes were derived. Rhetorical strategies that frequently occurred alongside specific narrative tropes were noted. Examples from the corpora were assigned to each code in order to further illustrate.

## 3. Results

Of the final codebook’s list of narrative tropes, five patterns represented more than half of the coded items: “Corrupt Elites,” “Vaccine Injury,” “Sinister Origins,” “Freedom Under Siege,” and “Health Freedom.” Of the final codebook’s list of rhetorical styles, four patterns represented just over 40% of all coded rhetorical items: “Think of the Children!” “Do Your Own Research,” “Heroes and Freedom Fighters,” and “Panic Button” (see Table 1). All nine of these codes appeared across all four major platforms from which data were sampled: YouTube, Twitter, Facebook, and Instagram. However, it should be noted that salience cannot be determined solely by the frequency with which a code appeared either within or across channels. In keeping with this study’s action research methodology, other factors, such as interaction with other narratives and rhetorics and subjective judgements of affective intensity, were taken into account when determining salience. In total, the results of our coding show that online, English-language, anti-vaccine, and COVID-denial content can be classified according to twenty-two narrative tropes and sixteen rhetorical strategies (see Table 2 and Table 3). The twenty-two narrative tropes can further be classified as directed toward five major types of antagonists: (i) the government, (ii) the medical establishment and political/economic elites; (iii) mainstream (and implicitly, pro-vaccine) society at large; (iv) an entirely unspecified “shadowy villain”; (v) the vaccine itself. Some additional unclassified antagonists form a miscellaneous category.

### 3.1. Narrative Tropes

Of the twenty-two narrative tropes coded, four key common narrative tropes were: “Vaccine Injury,” “Corrupt Elite,” “Heroes and Freedom Fighters,” and “Sinister Motives.” Each of these narrative tropes addressed a separate antagonist, representing all categories of antagonist—the Government/the Medical Establish/Media, Society at Large, a Shadowy or Unknown Villain, and the Vaccine Itself—except for “Misc./No Clear Antagonist.”

The narrative of “Corrupt Elite” was the most frequently coded narrative. This offered a standard populist appeal in which an innocent put disempowered “silent majority” suffer under the tyranny of a powerful and corrupt minority [87]. In a process of circular logic, the fact that the government, medical establishment, and high-profile cultural figures support such measures as lockdowns, masks, and vaccines is taken as sufficient evidence that these measures are untrustworthy. The narrative of a corrupt elite was very frequently seen with related but distinct codes. “Follow the Money,” for example, often co-occurred, offering financial motives for elite corruption. “Sinister Motives” offers malevolent and even supernatural explanations for elite missteps. The “Unaccountable Elites” narrative frames government and the medical establishment as immune to consequences from the bad or incompetent actions; it is motive agnostic, but argues that whatever their motives, elites bear no consequences for the mistakes and, therefore, are always to be mistrusted and disobeyed. These, along with other compatible narratives, point to a larger metanarrative that pits potential vaccine recipients against all of the social institutions working to provide them with a vaccine.

“Vaccine Injury” appeared to be the most salient of narratives, though not the most frequent. The code was a catch-all concept describing all negative outcomes (almost exclusively imagined or untrue) associated with taking a vaccine. Examples of this narrative rarely provided a clear causal link between vaccination and injury. Instead, it most frequently juxtaposed a claim to having been vaccinated alongside a claim to injury. These injuries were often vaguely described and only infrequently accompanied by claims to an actual diagnosis of malady. Messages conveying this narrative frequently used the “Panic Button” style of audio-visual rhetoric, in which images of needles, crying infants, unsettling sounds, or unattractive colors are employed to produce feelings of disgust and unease.

A third popular narrative, “Heroes and Freedom Fighters,” is compatible with populist framings, presenting anti-vaccine or COVID-denialist medical doctors (as well as chiropractors and naturopaths) as brave whistleblowers, risking their reputations and careers by speaking truth to power. This narrative often coincided with rhetorics of “Brave Truthteller,” “Sleeping Giants,” and “Health Freedom,” presenting the people organizing against public health measures as the moral equivalent of pro-democracy dissidents.

### 3.2. Rhetorical Strategies

Of the sixteen rhetorical strategies coded, four key, common rhetorical strategies were: (i) the “Brave Truthteller,” (ii) “Do Your Own Research (DYOR),” (iii) “Mountains and Molehills,” and (iv) “A Global Movement/Sleeping Giants”. Each of these rhetorical strategies was present across multiple categories of narrative antagonist. For example: the rhetorical strategy labeled “Brave Truthteller”—in which a speaker claims to be speaking a dangerous truth that “the establishment” or society at large is suppressing—was significantly present in all four categories of antagonists and even in those relatively few narratives with no clear antagonist.

Another common rhetorical strategy that is directed toward multiple protagonists is “DYOR” or “Do Your Own Research.” DYOR works by trying to empower the audience to develop their own bodies of evidence and methods of reasoning in order to reach a preordained conclusion. Sometimes, this leads inquiring minds to build their own arguments in favor of a predetermined position. Other times, DYOR offers vaccine denialists and skeptics a means to avoid answering questions or engaging in debate: they may simply demand that the interlocutor should do their own research. If the interlocutor reaches the correct conclusions (i.e., anti-vax conclusions), then they have done good research. If they still disagree, so the reasoning goes, their research must have been bad.

A third popular category is what we refer to as “Mountains and Molehills.” In this rhetorical strategy, vaccines’ risks and benefits are presented without a proper sense of proportion. Extremely small risks (e.g., negative reactions to vaccines) are framed as catastrophic and universal. Simple and far-reaching solutions (e.g., mask mandates) are presented as onerous and ineffective. The risk/reward calculus is thereby severely distorted. This strategy is used to seed doubt via emphasizing fringe or outlier cases.

Presenting COVID-19 vaccine resistance as a part of a global movement of “sleeping giants”—honest, everyday citizens who are on the cusp of rising up against an oppressive “global elite”—was a common rhetorical strategy that cut across narratives targeting all categories of antagonists. This rhetoric frames its accompanying narrative to suggest that the voices of ordinary people all around the world are articulating that narrative as a unified mass. Anti-vax and anti-public health movements are presented to be just the beginning of this groundswell. All over the world, so the trope goes, self-conceived “ordinary people” are ready to rise up and take back the power over their own lives. This rhetoric taps into the populist persuasive strategies and schemata outlined above, which frame the pure, ordinary people against the nefarious, evil elite. In some cases, populist rhetoric intersects with nationalist rhetoric, arguing that only a stronger state can save ordinary people from bad elites. In other cases, these populist frames intersect with anti-government resistance, framing elites as not only out of touch with the needs of the pure, ordinary people but actively working against them in tyrannical ways that warrant uprisings, revolution, armed resistance, or even, a new civil war.

## 4. Discussion

The scope of this study is intended to create a codebook of online English-language anti-vaccination narratives and rhetoric, so as to support government officials and civil society groups engaged in managing disinformation during the COVID-19 vaccination campaign. Further research into languages besides English tailored to address the regional cultural contexts that shade narratives and rhetoric are underway by members of this team, but beyond the scope of the present article. Local, national, and international governments, as well as civil society organizations, need to be prepared to manage the infodemic by promoting the timely dissemination of accurate information based on science and evidence, in particular to high-risk groups. This dissemination of accurate information should be both positive and defensive. That is, messaging campaigns must communicate both the latest in scientific understanding of COVID, its spread and prevention, and effective counter-messages against misinformation. The codes identified by this study offer governments and civil society groups a catalogue of anti-vaccine message styles that may assist in the latter efforts.

Pro-vaccine audiences, the vaccine hesitant, and the wholly agnostic can generally be addressed as a single group. Anti-vaccine belief holders, a much smaller subset of the overall vaccine hesitancy spectrum [89], however, must have counter-messaging tailored specifically for them. This comports with the distinction between “prophylactic” and “therapeutic” counter-messaging [90]. Evidence demonstrates that prophylactic exposure to counter-messaging would prompt hesitant, agnostic, and pro-vaccine audiences to develop their own counterarguments against misinformation and disinformation [91,92]. Furthermore, these audiences would not need to be warned against every type of misinformation or disinformation they might encounter. Exposure to counter-messaging against one dimension of a mis/disinformation campaign confers resistance to other dimensions associated with that topic of mis/disinformation [93,94,95]. This phenomenon is sometimes called the “blanket of protection” [96,97]. So, for example, an effective counter-message addressing exaggerated claims of vaccine injury can in theory be expected to confer some resistance to any other narrative trope or rhetorical strategy listed in this codebook.

The joint effects of targeted counter-messaging and blanket of immunity might best be mobilized through counter-messaging that addresses tropes and/or strategies that appear most frequently, and that are thematically linked to other denialist narratives and rhetoric. For example, the versatility of rhetorical strategies such as “Brave Truthtellers” may be both a cause and a symptom of their effectiveness; its versatility lends itself to a variety of narratives, while its repetition across narratives enhances its effectiveness through repetition. Counter-messaging that alerts audiences to the manipulative persuasion of the “Brave Truthteller” strategy will, therefore, be effective against a variety of misinformation and disinformation utilizing the strategy, as well as likely conferring further future resistance against the rhetoric and narratives that appear alongside it.

Similarly, “Do Your Own Research” is also a popular rhetorical strategy throughout conspiracy cultures, such as QAnon. QAnon disinformation networks have been shown to amplify anti-vaccination rhetoric and messaging [98], which raises important questions about the impact of overlapping persuasive rhetorical strategies in addition to amplification through hashtags or coordinated campaigns. By counter-messaging against transnetwork themes, a blanket of immunity may protect audiences from anti-vaccine and COVID-denialist themes that are not described in this codebook or have yet to emerge.

Counter-messaging to address audiences who already hold undesirable viewpoints (i.e., therapeutic counter-messaging) is a newer and less well-established process than its prophylactic counterpart [90,96,99]. A potential for backlash is always present in counter-messaging campaigns [100,101]. Additionally, while the so-called “backlash effect” (that is, the theory that factual counterargument entrenches false beliefs) has been credibly challenged [102], there is ample evidence that carelessly repeating false information can help spread it [103,104,105,106]. Therefore, public health messaging that addresses anti-vaccination audiences, already hardened in their beliefs, must be preceded by especially rigorous testing. This codebook is presently informing a series of tests to determine the efficacy of public messaging that addresses some of the narratives and rhetoric found in it. The results of that study are expected to be available in pre-print by early summer 2021.

## 5. Limitations and Future Directions

The present study has shed light on narrative and rhetorical patterns in anti-vaccine and COVID-denialist online media. However, the methods that made this study possible have also limited it in key ways. While the combined deductive/inductive coding process appeared to produce a saturation of codes, this cannot be definitively stated absent a corroborating study based on a large data sample. Such a study might be undertaken, using the present study’s codebook as a deductive point of departure for a big data text mining and topic modeling project. It is possible that such an expanded study might reveal unexplored dimensions of anti-vaccine and COVID-denialist positions.

By the same token, this study was conducted with the intent of helping to inform public health messaging. A follow-up study is currently underway, which utilizes several of the codes mentioned in this article to create public health counter-messages. Among other questions, this follow-up study tests the efficacy of emotionally based counter-messages relative to factually based counter-messages. It asks: is form indeed more salient than content to debunk health misinformation and disinformation? Here, more research is needed. Further work might also consolidate codes to supplement the “antagonist” categories offered here. Such a consolidation may help others to adopt these codes in independent public health messaging endeavors.

Finally, it is worth delving into the question of “why” these narratives and rhetorics hold such appeal. One might easily imagine a study of people holding anti-vaccine and COVID-denialist attitudes, which tests the salience of the narratives and rhetorics presented here. Such a survey could also address overlapping attitudes and worldviews to better ascertain the narratives indicated to believers by these tropes, and why such narratives and rhetorics are appealing.

## 6. Conclusions

This paper reports on the qualitative classification of online, English-language anti-vaccination rhetoric about a COVID-19 vaccine. Our analysis of sixteen persuasive rhetorical strategies and twenty-two anti-vaccination messages directed toward specific antagonists is necessarily descriptive at this stage. We hope this work inspires additional empirical research to help illuminate the following questions: which of these rhetorical strategies and messages are most encountered online and for which demographic groups? Which of the messages carries the most persuasive appeal? How can persuasive rhetorical strategies be effectively countered in online spaces? How might different narratives and rhetorics appeal to audiences in different countries, different demographics and subcultures, and in different languages? Given the rise in populist movements across many countries, it seems possible that similar antagonisms would be articulated in anti-vaccine and COVID-denialist media content. However, given the highly contextual and referential qualities of memes and other social media content, these antagonisms could reasonably be expected to assume significantly different expressive forms. To avoid the risk of blowback, it is essential that similar coding studies and counter-message testing be undertaken prior to the launch of public health campaigns addressing anti-vaccine and COVID-denialist mis- and disinformation.

## Figures and Tables

**Table 1:** Top codes by prevalence.

Narrative Trope	Explanation	Prevalence (Percentage of Final Codebook)
Corrupt Elites	Populist framing of a righteous majority versus corrupt elite. Elite perceived as forcing lockdowns and health practices for their own financial gain (e.g., big pharma) and/or power.	20.4%
Vaccine Injury	A catch-all for all of the harms the vaccine can do to you, from physical deformities to mental illness to microchips that violate your autonomy/privacy.	12.1%
Sinister Origins	The people who intentionally created the COVID vaccine are shadowy and suspicious. Geopolitical powers and intelligence agencies are likely implicated.	10.4%
Freedom under Siege	Common rights such as speech, assembly, or autonomy are being stripped from you! This claim attempts to hijack feelings of protection, vulnerability, and the sacred.	9.3%
Health Freedom	Frames public health as a matter of individual freedom rather than collective responsibility. “My body, my choice” misapplied to vaccines. Tied to religious freedom/freedom of speech.	7.8%
**Rhetorical Strategies**	**Explanation**	**Prevalence**
Think of the Children!	Frames this as an advocacy campaign for protecting children. Uses emotionally affecting (manipulative) images of cute children.	16.1%
Do Your Own Research	States a conclusion and then urges the reader to research the reasons why. Visual clues lead to building arguments in favor of a predetermined anti-vax position.	8.4%
Speaking Truth to Power	Doctors, nurses, and other professional “experts” speaking against COVID alarmism are brave whistleblowers, courageously bringing truth to the people.	8.1%
Panic Button	Audio and visual cues intended to spark alarm, fear, or disgust, such as ominous music and images of needles or malformations.	7.2%

**Table 2:** Rhetorical strategies.

1. Absurd!
Rhetoric that holds up public health practices and cultural expressions of care/anxiety over COVID-19 to ridicule. This includes ridiculing both experts and laypeople, sometimes through misrepresentation (see “Mountains and Molehills”). Key to this rhetorical strategy is an overall tone of mockery and/or contempt.
**2. Appropriating Feminism and/or Womanhood**
Anti-vaccination messages that appropriate the language and values of feminism, such as claiming that vaccine resistance is the positive moral equivalent of advocating for reproductive rights; also, sometimes appropriating themes of femininity, the stereotypes of maternal wisdom and nurturance.
**3. Brave Truthteller**
This strategy frames the speaker as brave for publicly voicing their anti-vaccine opinions, despite the potential for public backlash, parenting judgment, or criticism from supposed experts. This strategy celebrates vaccine resistance by depicting its messengers as heroic in their stand against the establishment, akin to a whistleblower standing up to corruption. Sometimes, this takes the form of the speaker themselves claiming he or she is brave; other times, someone else’s bravery is highlighted. In vaccine-hesitant and -resistant spaces, the bravery often pertains to standing up to others’ ridicule, or to the implication that one is a bad parent.
**4. DYOR (Do Your Own Research)**
This approach often states a conclusion contrary to mainstream beliefs or scientific consensus, and then urges the audience to research the reasons why the conclusion is correct. This leads inquiring minds to build their own arguments in favor of a predetermined position. Other times, this strategy is deployed to avoid answering questions or engaging in debate. The implication is that if audiences reach the correct conclusions (i.e., the ones the speaker asserted), then they have done good research. If they disagree, their research must have been bad.
**5. Epic Significance**
The struggle against vaccination is framed as one of global, historical, or even mythic proportions. Hyperbolic rhetoric and superlatives are used to convey that this threat is profound enough to change the world, to enshrine the power of a corrupt elite—or to imperil the most vulnerable among us (children). In addition to the exaggeration of the threat posed by vaccines, this strategy positions the audience as capable of, or even obligated to, participate in this epic quest for justice.
**6. A Global Movement/Sleeping Giants**
Rather than inflating the threat of the vaccine to epic proportions, this strategy inflates the anti-vaccine movement itself. The voices of ordinary people all around the world are depicted as speaking as one, a unified, grassroots groundswell against evil. Sometimes anti-vaccine and anti-public health movements are framed as just the beginning of a groundswell that addresses other conspiracies. This strategy employs a populist frame that all over the world, good ordinary people (“just plain folks”) are ready to rise up and take back the power over their own lives.
**7. Health Freedom**
This strategy frames public health as a matter of individual freedom rather than collective responsibility. Sometimes, this even borrows from the language of women’s reproductive rights, re-appropriating concepts such as “my body, my choice” to vaccines. This is sometimes related to religious freedom (vaccines) or freedom of speech (anti-mask).
**8. Hijacking Familiar Formats**
In general, memes work by using a familiar format to make a new joke or prove a new point. Rhetorical strategies that hijack formats adopt popular formats that are associated with harmless humor and use that familiarity to lower our emotional and cognitive defenses. With its audiences’ critical defenses down, it then presents its manipulative message. Beyond memes, this also applies to TV, film, and game formats that are appropriated for propaganda. This strategy also includes “hashtag hijacking,” where vaccine misinformation will be tagged with hashtags relating to less controversial issues and groups, such as #blacklivesmatter or #americancancersociety. Hashtag hijacking is particularly effective for exposing newcomers to anti-vaccine messages.
**9. Intersecting Social Justice**
Rhetoric similar to this frames vaccine skepticism alongside social justice issues or frames vaccine skepticism itself as a social justice issue in its own right. Sometimes, it may invoke systemic racism or the abuse of minorities by the police to imply that the medical establishment is similarly racist. It may frame its argument more generally, too, presenting vaccine resistance as a matter of either civil rights or religious conscience. It may describe non-vaccinators as “health minorities” or “dissidents.”
**10. Lovebombing**
Allies and fellow “truth-tellers” are showered with affirmation, accolades, validation, and compliments. This often includes emotive videos or images celebrating allies for their cause as heroes or persecuted saints (see also: “Sleeping Giants”).
**11. Mountains and Molehills**
Risks and benefits to vaccines are presented with intentionally distorted proportions. Extremely small risks (e.g., negative reactions to a vaccine) are framed as catastrophic and universal. Simple and far-reaching solutions (e.g., mask mandates) are presented as onerous and ineffective. This strategy distorts the risk/reward calculus of vaccines in order to seed doubt, often by emphasizing fringe or outlier cases of vaccine injury.
**12. Panic Button**
A common rhetorical technique that uses audio and visual cues intended to spark alarm, disgust, confusion, squeamishness, anxiety, or dread in audiences. Ominous music can be used to indicate that viewers should be worried or mistrustful about what is shown to them. Images of hypodermic needles, malformations purportedly caused by vaccines, or forced vaccinations are depicted in ways that evoke fear and/or disgust.
**13. People are Saying**
This strategy states or implies that “many” people feel a certain way, evoking a social norm against vaccination. The strategy depicts those who agree with the speaker as good people, and those who disagree as fearful conformists. It may imply that evidence exists simply because other people are allegedly saying it, even though there is no actual evidence presented. Otherwise, it may rely on testimonies, first-hand accounts that usually emphasize emotion over facts, and may or may not actually be true.
**14. Question Begging**
A technique that poses questions designed to set up a narrative, as opposed to asking questions for objective journalistic purposes. This strategy asks a series of questions that lead to a specific anti-vaccine answer, while framing the conversation as objective and inquisitive.
**15. Think of the Children!**
This rhetoric suggests that anti-vaccine advocacy is not about what activists want for themselves, but rather what is best for children. Arguments are framed so as to position children’s exaggerated physical vulnerability and moral purity as the decisive factor in assessing risk. It often uses emotionally affecting (manipulative) images of cute children.
**16. Trappings of Authority**
Often a visual rhetoric, this strategy uses symbols of authority and expertise to give added weight to an argument. A speaker might be in an office full of books. They might be in a doctor’s office. They might be expensively dressed. The interviewer or director might refer to them with exaggerated deference. Sometimes, their credentials are presented as if they were very impressive but, when examined more closely, are spurious or over-inflated.

**Table 3:** Narrative tropes (organized by primary antagonist).

**Antagonist: Government/Establishment and Elites:** Narratives 1–8 are framed in such a way as to villainize experts, authorities, and figures of cultural influence. These “elites” consist of groups such as the medical establishment, governments, media, and press.
**1. 1984**
This narrative depicts the COVID pandemic and all public health measures associated with it as the final few steps toward a maximally repressive global government. It presents a “domino theory,” in which free speech, freedom of religion, and freedom of travel will soon be abolished. Every time a new public health directive has been passed, it says, many vaguer, but far worse, oppressions are sure to come next.
**2. Alarmist Authorities**
This narrative presents a distorted pattern of events in which authorities’ warnings and measures against COVID are overblown. (see Fluffing the Curve and Follow the Money)
**3. Censored!**
Digital platforms and social media are portrayed as actively engaged in “censoring” advocates of “health freedom.” This is often framed as a David vs. Goliath scenario where powerful companies conspire against brave individuals speaking truth to power. This is described in the language of a grave injustice.
**4. Corrupt Elite**
This narrative is a standard populist appeal. The world can be divided into a corrupt elite and a righteous majority. The corrupt elite is on the side of lockdowns and mandatory masks/vaccines. The fact that the elite favors these lockdowns, masks, and vaccines is taken as more than sufficient evidence that they should not be trusted. So the reasoning goes: the elites must be corrupt, because they are pushing an untrustworthy and potentially dangerous medicine.
**5. Fluffing the Curve**
This narrative argues that officials are misrepresenting the numbers of COVID injuries and deaths, or that doctors are somehow incentivized to report more deaths. Perhaps they are doing so to ensure profits (see “Follow the Money”), or perhaps to instill fear and control (see “1984,” “Sinister Motives”). This category also includes “apples to oranges” comparisons of patient categories, different diseases’ mortality rates, vaccinated vs. unvaccinated health outcomes, and more.
**6. Follow the Money**
This narrative paints the COVID pandemic as an unprecedented opportunity for corporate looting and medical profiteering. Additionally, anything that points to more robust public health initiatives is almost certainly a set-up for crony handouts and panic-driven marketing. There is big money in medicine, this narrative says, and for media giants, there is big money in making people “panic-watch” and “doomscroll.” These are stories in which powerful men will do whatever it takes to compete and aggrandize their wallets and ego—whether it means lying, neglect, withholding care or resources, or plain out killing. There is a specific sub-category that describes claims made against Anthony Fauci regarding supposed fraud. Most famous is the “HIV Scandal” involving a series of vague accusations of silencing patients, academics, and scientists to uphold a Ponzi scheme related to HIV treatment protocols [88].
**7. Freedom Under Siege**
This narrative paints a story in which common rights such as speech, assembly, or possession of some entitled object are being stripped from citizens. This claim attempts to hijack feelings of protection, vulnerability, and the sacred. Can also be framed with the key words, “Religious and Philosophical Exemption.”
**8. Unaccountable Elites**
These narratives are framed around the assumption that doctors, politicians, and the media will never have to account for their lying or incompetence. So the story goes: if they have no skin in the game, then why should we believe a word they say?
**Antagonist: Society at Large:** Narratives 9–12 pit anti-vaccine advocates and COVID denialists against society in general or specific elements of it, such as our public political discourse or areas where racial disparities are acutely felt.
**9. Heroes and Freedom Fighters**
Here, doctors (and “doctors”) speaking out against vaccine injury or COVID alarmism are brave whistleblowers, acting at tremendous personal and professional risk to bring the truth to the people. The people protesting public health measures are painted as the moral and ideological equivalent of Soviet dissidents, the founding fathers, and the Arab Spring all rolled into one. This narrativizes the “Brave Truthtellers” rhetorical strategy by imbuing it with specific protagonists and struggles.
**10. Erasing POC**
This narrative argues that people of color are shut out of public debate over vaccination, that their voices are dismissed, or they are tokenized and only deployed when it is convenient for the white and powerful. It might also argue that people of colors’ rights to “health freedom” or their experiences of “vaccine injury” are invisible due to systemic racism in the medical system. It usually accompanies tropes such as “Racist Medicine” or “POC Injury.” It is an example of how effective anti-vaccine narratives can be essentially correct, but still point toward false and damaging conclusions.
**11. Racist medicine**
This narrative points to the real history of medical abuse of minorities in the US and elsewhere and implies that minorities should, therefore, mistrust what they hear about COVID and vaccines. Usually, no specific threat or conspiracy is articulated. The history is described and the connection with the present day is left implicit, but clear (see also: Intersection with Social Justice, Erasing POC, POC Injury).
**12. “You made it political!”**
This frames the conflict between vaccination and non-vaccination as a partisan political issue. On one hand, it might state that pro-public health voices are the ones making this political, when it is actually a matter of common sense, religious freedom, or personal choice. On the other hand, this narrative category might take an explicitly partisan tone, for example arguing that former President Donald Trump was heroically battling big pharma and a corrupt elite.
**Antagonist: Shadowy Villain:** Narratives 13–14 do not offer a specific villain, but implicate an extremely powerful and mysterious agent whose means and motives are unknown—perhaps beyond comprehension. Conspiracy theories that verge on the supernatural often framed their antagonist in these terms. These demonstrate that narratives can be based around an absence or unarticulated mystery (see literature review).
**13. Chinese Virus**
These stories claim with absolute certainty while lacking in substantive proof that the virus was created or leaked from the Wuhan lab in China. These tropes are distinct from legitimate inquiry into a possible “lab-leak hypothesis,” because of the narratives that they indicate. Sometimes, those narratives claim that a virus cannot mutate that quickly, or that COVID is a powerful bioweapon and the idea that we can easily stop it with masks or a vaccine is laughably naïve. These narratives are highly compatible with long-existing anti-Asian stereotypes as a sinister “enemy within” Western countries.
**14. Sinister Motives**
The people behind the COVID vaccine are described as shadowy and suspicious. Geopolitical powers, pharmaceutical corporations, and intelligence agencies are likely implicated.
**Antagonist: The Vaccine Itself:** Narratives 15–19 focus on the harm they imagine a COVID vaccine will inflict. Unless tied to another narrative or rhetoric specifying additional antagonists or personifying the vaccine, these narratives offer an antagonist that is impersonal and without motive.
**15. The Perfect Family**
These narratives are often framed around anecdotes of supposed vaccine injury. Children are presented as perfect angels, baby geniuses, junior Olympians, etc. Parents are presented as bursting with pride, ready for a smooth, normal, American (or English or Australian or w/e) life. Then came the vaccine, and its injury. Then came the never-ending tribulations. The dream is long dead.
**16. POC Injury**
This narrative states that ethnic minorities have congenital conditions which allopathic medicine does not properly consider during the development of treatments and vaccines. One example is the claim that African Americans, particularly boys, have stronger immune systems that are more reactive to vaccines. While the coding team did not encounter similar messages targeting women of any race, it seems possible that women’s higher rates of autoimmune disorder, and historic mistreatment in medicine, could underpin similarly pseudoscientific theories (see also “Racist Medicine”).
**17. Rushed Vaccine**
These narratives say that the COVID vaccine has been rushed to market without proper testing, that it could not have gone through trustworthy safety protocols, and that the public cannot trust that it will be safe.
**18. Unknowable Dangers**
These narratives assume that we should apply the precautionary principle to dangers associated with preventing COVID (i.e., vaccines) but not to COVID itself (e.g., the danger is overblown, go to the pub!) (see also: Mountains and Molehills). This is distinct from the Vaccine Injury narrative, as it focuses on vague potential future outcomes, whereas Vaccine Injury focuses on specific, and often present-day, claims of injury.
**19. Vaccine Injury**
A catch-all term for all the bad things vaccines can do to you, with no legitimate causal link required. Extremely common.
**Miscellaneous/No Clear Antagonist:** Narratives 20–22 either did not present a clear antagonist or were not consistent enough in their imagined antagonists to effectively classify.
**20. All-or-Nothing**
These narratives cast their heroes and villains as either all trustworthy, good, and “on the right side” or else dangerously misguided, stupid, or evil.
**21. Imminent Threat**
Narratives of this sort warn their audience that “time is running out,” and something terrible is either happening or about to happen very soon. This threat could be specific (e.g., a law being debated that would mandate vaccines for public school attendance), or it could be vague (e.g., the end of America). The warning is very frequently accompanied with some call to action, such as calling your congressman or evangelizing in favor of anti-vax messages.
**22. Overblown Risk**
These narratives dismiss risks associated with COVID as overblown. They sometimes misuse statistics to reach this conclusion, such as comparing high-risk populations’ flu mortality rates to low-risk populations’ COVID mortality. Most often, these narratives center around an emotionally dismissive claim of others’ alarmism. This is distinct from the Alarmist Authorities code, as it addresses a more general cultural alarmism that may originate in not-elite sources.

## Data Availability

The data presented in this study are available on request from the corresponding author. The data are not publicly available due to concern for the privacy of the subjects whose public social media posts were used in the codebooking process.
